# The Importance of Relevance: Willingness to Share eHealth Data for Family Medicine Research

**DOI:** 10.3389/fpubh.2018.00255

**Published:** 2018-09-04

**Authors:** Gillian Bartlett, Brenda Macgibbon, Analia Rubinowicz, Cecilia Nease, Martin Dawes, Robyn Tamblyn

**Affiliations:** ^1^Family Medicine, McGill University, Montreal, QC, Canada; ^2^Mathematics, Université du Québec à Montréal, Montreal, QC, Canada; ^3^Joan C. Edwards School of Medicine, Marshall University, Huntington, WV, United States; ^4^Family Medicine, University of British Columbia, Vancouver, BC, Canada; ^5^Epidemiology and Biostatistics, McGill University, Montreal, QC, Canada

**Keywords:** family practice, community health services, secondary data analysis, patient participation, informed consent

## Abstract

**Objective:** To determine the proportion of family medicine patients unwilling to allow their eHealth data to be used for research purposes, and evaluate how patient characteristics and the relevance of research impact that decision.

**Design:** Cross-sectional questionnaire.

**Setting:** Acute care respiratory clinic or an outpatient family medicine clinic in Montreal, Quebec.

**Participants:** Four hundred seventy-four waiting room patients recruited via convenience sampling.

**Main Outcome Measures:** A self-administered questionnaire collected data on age, gender, employment status, education, mother tongue and perceived health status. The main outcome of was self-reported relevance of three research scenarios and willingness or refusal to share their anonymized data. Responses were compared for family practice vs. specialty care patients.

**Results:** The questionnaire was completed by 229 family medicine respondents and 245 outpatient respondents. Almost a quarter of all respondents felt the research was not relevant. Family medicine patients (15.7%) were unwilling to allow their data to be used for *at least one* scenario vs. 9.4% in the outpatient clinic. Lack of relevance (OR 11.55; 95% CI 5.12–26.09) and being in family practice (OR 2.13; 95% CI 1.06–4.27) increased the likelihood of refusal to share data for research.

**Conclusion:** Family medicine patients were somewhat less willing to share eHealth data, but the overall refusal rate indicates a need to better engage patients in understanding the significance of full access to eHealth data for the purposes of research. Personal relevance of the research had a strong impact on the responses arguing for better efforts to make research more pertinent to patients.

## Introduction

Research in the field of primary care is rapidly expanding, especially with an increasing emphasis on the role of family medicine for improving quality of health care (1, 2). The concomitant proliferation of health information technology, with clinical data being captured electronically (eHealth data), provides an invaluable resource for research and clinical care (3, 4). The debate, however, continues around secondary access to eHealth data for the purposes of research (5–8).

There is ongoing discussion about whether patients must, need, or want to provide explicit consent for this activity (9–12). The use of written informed consent for access to data for both clinical and research purposes creates significant sources of selection bias (13–15). People with less education, lower income and poor continuity of care are less willing to provide informed consent or may not even be approached to provide consent (16). Typically, this is characteristic of the patients in family medicine that are the most vulnerable, have restricted access to care and have poorer health outcomes (17). It is important to note that even a small proportion of people being unwilling to share their eHealth data can create significant biases given how these data used (16).

In addition, primary care clinics represent a very different patient population than those typically seen in acute care and research-oriented institutions (18). The patients who seek out primary care are generally not as ill and are less familiar with health research procedures and objectives (19). eHealth data is increasingly available and would be an excellent resource for research that can improve health care delivery and optimize patient outcomes in this context (20). Patients who are not exposed to health research may not see the value of researchers having access to their eHealth data. The purpose of this study was to determine what factors might make family medicine patients in primary care unwilling to allow their eHealth data to be used for research purposes and to evaluate the impact of patient characteristics and relevance of the research topic on this lack of willingness.

## Methods

For this cross sectional study, participants were recruited using convenience sampling of consecutive attendees in waiting rooms at a family medicine clinic (Montreal General Hospital Family Medicine Clinic) and an acute care outpatient respiratory clinic (Montreal Chest Institute) in Montreal, Quebec. All patients were eligible provided they were physically and mentally capable of completing the questionnaire. Research assistants trained in recruitment and interviewing techniques approached people while they were waiting for their clinic visit and gave a brief explanation of the project. For people who appeared to have difficulty with the documents, the research assistant approached them and offered to assist.

A self-reported questionnaire (French and English) containing three research scenarios for population studies was provided to participants. A preamble explained that the questionnaire was anonymous and explained how data collected for health research was stripped of identifying information to protect people's privacy and that the three research scenarios used this type of data. The research scenarios were modified, with permission, from the Canadian Institutes for Health Research Report “Secondary Use of Personal Information in Health Research” (21).

Scenario 1evalutates drug costs for the elderly:

“This study used prescription drug data linked with hospitalization data to examine the effect of a policy requiring elderly and welfare patients in Quebec to pay a greater share of their prescription drug costs under the provincial drug plan. The study showed that the policy resulted in these patients using fewer medications and needing more admissions to hospital. The study findings resulted in immediate change to the provincial drug insurance policy.”

Scenario 2 examines the safety of breast implants:

“Surgical records were used to identify 25,000 women in Ontario and Quebec who had received breast implants for cosmetic reasons. Information about the women and their surgical procedures taken from physician and hospital records were linked by Statistics Canada with information on deaths or diagnosis of cancer. The researchers used this information to determine whether women who had breast implants were at a greater risk of getting particular cancers or dying than women in the general public.”

Scenario 3 determines if clinicians were treating heart disease according to guidelines:

“Some drugs used to correct irregular pumping of the heart can, in some people, cause serious heart problems themselves. Professional practice guidelines recommend what types of drugs should be prescribed to avoid this problem. In this study, prescription drug data were linked with hospital records to determine whether physicians in the province were following these guidelines and how often not following the guidelines resulted in an increase in the number of patients suffering from heart rhythm problems.”

In response to each scenario, patients were asked, “Would the findings from this study be of interest to you, a member or your family or someone you care about?” Willingness to share anonymized eHealth data for research was measured for each scenario based on common models of consent for the use of de-identified data other than written informed consent. These models included no consent required; notification of the use of the data but no consent required; no consent required but the patient can “opt out” from being included in the database; and no use of the data permitted. To measure willingness, people were asked to “Please indicate how you feel about your health information being used for this research by checking one of the four statements below with which you most strongly agree.” The statements were: (1) they should use my health information if they need it (no consent); (2) they should use my health information but I want to know when this is being doing (notification model); (3) they should use my health information but I should have the option of saying no (opt-out model); and (4) my health information should not be used.

The questionnaire collected information on patient characteristics that have been previously shown to be associated with consent: age, gender, income and education levels, marital status, primary language and perceived health status (13). The perceived health status was assessed using a visual analog scale (VAS) indicating general health with 0 indicating “worst imaginable state of health” and 100 indicating “best imaginable state of health.” This study was carried out in accordance with the recommendations of Tri-council Policy. The protocol was approved by the McGill University Health Centre Research Ethics Board Ethics. As all information was collected anonymously, written informed consent was not required in accordance with the requirements of the ethics committee.

### Statistical methods

Chi-squared tests and Fisher exact tests (when necessary) were conducted to test whether the patient characteristics were different between the two clinics for all variables except for the average perceived health score where a Student *t*-test for unequal variances and a non-parametric Wilcoxon two-sample test were used. The main outcome sought was a patient's unwillingness to share eHealth data for research purposes. This variable was coded with 1 corresponding to unwillingness to share data and 0 to yes, unsure, missing. We chose this coding to reflect that removal or a definite “no” is more significant in eHealth data accrual than a lack of response or uncertainty. Relevance was coded with 0 (reference group) corresponding to yes and 1 to no, unsure or missing. To determine the effect of the patient characteristics, the clinic and the relevance of the scenario presented on the unwillingness of the patient to share eHealth, the data were included in a clustered logistic model, clustered by scenario using an auto-regressive correlation structure (as the scenarios did not vary in order).

To determine whether the interaction between irrelevance and unwillingness to share data were different between the two patient populations, that is, those at the family medicine clinic compared to those at the acute care outpatient clinic, the two-way interactions between these two variables were tested using Chi-squared tests and Fisher exact tests whether these interactions were significantly different between the samples for each scenario (SAS version 9.2).

## Results

There were 229 questionnaires completed at the family medicine clinic and 245 at the acute care outpatient clinic for a total of 474. As summarized in Table 1, family medicine respondents were more likely to be employed (*p*-value = 0.02), to be English speaking (*p*-value < 0.0001) and have a higher perceived health status [*p*-value < 0.0001 (*t*-test), *p*-value < 0.006 (Wilcoxon test)]. Other patient characteristics were not significantly different between the two groups (*p*-value > 0.05). These patient characteristics are summarized in **Table 3**.

**Table 1:** Patient characteristics for respondents from two clinics.

	**Family medicine (*n* = 229)**	**Outpatient acute care (*n* = 245)**
Proportion female	128 (55.9%)	137 (55.9%)
Proportion completing questionnaire in French	73 (31.9%)	131 (53.5%)
Proportion employed	142 (62.0%)	126 (51.4%)
Proportion with college degree or less	184 (80.3%)	208 (84.9%)
Proportion younger than 50 years	119 (52.0%)	126 (51.4%)
Average perceived health status (scale 0–100 with 100 being best)	80.4 (SD: 14.3)	73.7 (SD: 19.7)

Overall, 36 family medicine patients (15.7%) were unwilling to allow their data to be used for *at least one* scenario vs. 23 in the outpatient clinic (9.4%). A similar number for each clinic were unwilling to have their data used for *any* research scenarios (3.4% for family medicine and 4.1% for the acute care outpatient clinic) (Table 2). Almost a quarter of all respondents felt the research was not relevant to them (Table 3).

**Table 2:** Proportions who refused to share data among respondents from two clinics.

	**Refusal to share data family medicine (*n* = 229)**	**Refusal to share data outpatient acute care (*n* = 245)**
Scenario 1: Impact of Prescription Drug Co-Pay Policies on Health	22 (9.6%)	18 (7.4%)
Scenario 2: Association of Breast Implants with Cancer Risk	26 (11.4%)	17 (6.9%)
Scenario 3: Adherence to Clinical Guidelines for Cardiovascular Disease Treatment	12 (5.2%)	13 (5.3%)

**Table 3:** Proportion who found the research to be not relevant among respondents from two clinics.

	**Research not relevant family medicine (*n* = 229)**	**Research not relevant outpatient acute care (*n* = 245)**
Scenario 1: Impact of Prescription Drug Co-Pay Policies on Health	87 (38.0%)	94 (38.4%)
Scenario 2: Association of Breast Implants with Cancer Risk	93 (40.6%)	103 (42.0%)
Scenario 3: Adherence to Clinical Guidelines for Cardiovascular Disease Treatment	74 (32.3%)	88 (35.9%)

In the clustered logistic regression model, lack of relevance (OR 11.55; 95% CI 5.12–26.09) and being a family medicine patient vs. an acute care outpatient (OR 2.13; 95% CI 1.06–4.27) increased the likelihood of a patient's refusal to share data (Table 4).

**Table 4:** Odds ratios with 95% confidence intervals (95% CI) for factors impacting unwillingness to have e-health data used for research.

	**Odds ratio for refusal to share date**	**95% CI**	***P*-value**
Research not relevant	11.55	5.12–26.09	< 0.0001
Family medicine clinic vs. acute care	2.13	1.06–4.27	0.03
**PATIENT CHARACTERISTICS**			
Male vs. female	1.16	0.62–2.14	0.64
French vs. English speaking	1.51	0.80–2.87	0.21
Retired or unemployed vs. employed	1.56	0.79–3.08	0.20
University education or higher vs. college or less	0.71	0.26–1.92	0.50
50 years or older vs. 49 years or younger	0.82	0.41–1.66	0.59
Perceived health status	1.00	0.84–1.18	0.96

No significant differences were found in the interaction between lack of relevance and unwillingness to share data in the two groups of patients for scenarios 1 (drug costs) and 3 (heart disease) as measured using chi-squared tests and Fisher exact tests (when necessary) (*p*-value = 0.05). For scenario 2 (breast implant safety) acute care outpatients and those from the family medicine clinic significantly differed on the interaction between lack of relevance and unwillingness to share data (*p*-value = 0.03). The difference in the percentage of patients in each clinic who were unwilling to share data in scenario 2, when they found the scenario *relevant*, was not significant [2 (1.5%) of family medicine patients vs. 3 (2.1%) in the outpatient clinic]. The difference in the percentages who were unwilling to share data in scenario 2 when they found the scenario *not relevant* was significant [24 (25.8%) family medicine patients vs. 14 (13.6%) in the acute care outpatient clinic, *p*-value = 0.03]. The same tendency was found for the first and third scenarios, but the differences were not significant: for scenario 1, 20 (23.0%) family medicine patients vs. 13 (13.8%) acute care outpatients (*p*-value = 0.11) and for scenario 3, 10 (13.5%) family medicine patients vs. 8 (9.1%) outpatients (*p*-value = 0.37).

## Discussion

Family medicine patients were more likely to refuse to contribute their de-identified eHealth data for research purposes. Considering that the data was de-identified and did not require any further explicit participation from the patients, there was an important proportion of patients from both clinics who refused to allow access to their data for the purpose of health research, although the personal relevance of the research had a strong impact on the responses. The research scenarios provided were selected to be appropriate for the family medicine context; however, many patients did not perceive the research to be relevant to them or anyone they knew. While this supports our contention that exposure to health research is not the norm for patients in family medicine, it may also be related to the family medicine patients being healthier. Interestingly, the interaction for the breast implant safety study being the only statistically significant interaction, with family medicine participants less likely to share data, points to a need for a rigorous exploration of the issues touching on participation in research. This was the only scenario that was gendered and this is particularly concerning as other studies have found women less likely to participate in health research (16). Our findings in general, and this one in particular, argue for better efforts to make family medicine research more pertinent to patients including. This would be important to also address this in clinical education and training programs.

While we did find higher potential participation rates than researchers who more specifically investigated the effects of traditional informed consent on access to medical records who reported participation rate of less than 10% (22), our population was more educated than expected. This is a concern when there are higher refusal rates for subgroups such as older women or patients with mental health concerns (23, 24). We did not assess mental health and other researchers have found higher refusal rates for subgroups even when looking at authorization (notification model) (23). As our study was a self-reported questionnaire and focused on an unwillingness to share data, we cannot draw conclusions on what might have made participants take this decision, therefore the reasons for lack of relevance and refusal should be investigated further.

## Conclusion

To enhance research capacity, it will be critical to address the lack of research awareness and perceived relevance of research for patients in our family practice settings. In the context of secondary use of eHealth data, family medicine clinicians and researchers need to commit to a clear strategy that will enable this rich resource to improve delivery of care and clearly indicate the value and relevance of research for family medicine patients. Not only do we need to agree on the most appropriate, scientifically rigorous and ethically sound mechanism for access to the data, we must also become better self-promoters of research while advocating for appropriate access to eHealth data. To accomplish this, our knowledge translation and dissemination strategies need to target the important, and often ignored, stakeholder group of family medicine patients.

## Author contributions

GB conceived of the study objective and lead the oversight of the research project including writing of the manuscript. BM provided expert advice and supervision for the statistical analysis. AR and CN were students who assisted with analysis of the data, writing and editing of the manuscript. MD was the clinical lead who participated in the developing the study design, facilitating recruitment of participants and critical input into the manuscript. RT provided critical input in the development of the questionnaire, study design as well as revision of the manuscript.

### Conflict of interest statement

The authors declare that the research was conducted in the absence of any commercial or financial relationships that could be construed as a potential conflict of interest.
